# Targets for chimeric antigen receptor T-cell therapy of acute myeloid leukemia

**DOI:** 10.3389/fimmu.2022.1085978

**Published:** 2022-12-20

**Authors:** Christopher Schorr, Fabiana Perna

**Affiliations:** ^1^ Department of Medicine, Indiana University School of Medicine, Indianapolis, IN, United States; ^2^ Department of Biomedical Engineering, Purdue University Weldon School of Biomedical Engineering, West Lafayette, IN, United States

**Keywords:** CAR (chimeric antigen receptor) T cells, AML – acute myeloid leukaemia, target antigen, clinical trial, efficacy, safety

## Abstract

Acute Myeloid Leukemia (AML) is an aggressive myeloid malignancy associated with high mortality rates (less than 30% 5-year survival). Despite advances in our understanding of the molecular mechanisms underpinning leukemogenesis, standard-of-care therapeutic approaches have not changed over the last couple of decades. Chimeric Antigen Receptor (CAR) T-cell therapy targeting CD19 has shown remarkable clinical outcomes for patients with acute lymphoblastic leukemia (ALL) and is now an FDA-approved therapy. Targeting of myeloid malignancies that are CD19-negative with this promising technology remains challenging largely due to lack of alternate target antigens, complex clonal heterogeneity, and the increased recognition of an immunosuppressive bone marrow. We carefully reviewed a comprehensive list of AML targets currently being used in both proof-of-concept pre-clinical and experimental clinical settings. We analyzed the expression profile of these molecules in leukemic as well normal tissues using reliable protein databases and data reported in the literature and we provide an updated overview of the current clinical trials with CAR T-cells in AML. Our study represents a state-of-art review of the field and serves as a potential guide for selecting known AML-associated targets for adoptive cellular therapies.

## Introduction

Acute myeloid leukemia (AML) is an aggressive, rapidly progressing hematological malignancy. The 5-year overall survival rate of AML is only 27%, accounting for approximately 2% of all cancer deaths in the United States (1). While Chimeric Antigen Receptor (CAR) T-cell therapies have shown remarkable success in the treatment of acute lymphoblastic leukemia (ALL), demonstrating an impressive 82% response rate in relapsed & refractory B-cell ALL, developing CAR T-cells for AML has been challenging largely due to the lack of specific and suitable target antigens, complex clonal heterogeneity, and an increased recognition of immunosuppressive regulators in the bone marrow microenvironment. As a result, there is no formal FDA approval for CAR T-cell therapy in AML thus far.

CARs are synthetic receptors that retarget and reprogram T cells (2). Unlike the physiological T-cell receptor, which engages HLA-peptide complexes, CARs bind to native cell surface molecules without requiring antigen processing or HLA expression for tumor recognition (3), two mechanisms that promote immune cell evasion (4). An ideal CAR T-cell target is a cell surface protein that is highly expressed in all patients and by all malignant cells within the patients, including cancer stem cells. Surface targets that play a critical role in the pathogenesis of AML are more likely to block and challenge cancer cell mechanisms of survival and progression. As importantly, an ideal CAR T-cell target should not be expressed or very minimally expressed in normal tissues of the body including normal hematopoietic stems cells (HSCs) (5, 6).

In this manuscript, we carefully and systematically reviewed the characteristics of all molecules that have been proposed thus far as potential target antigens for CAR T-cell therapy of AML, both in pre-clinical and clinical settings. We describe the expression profile of these molecules in leukemic and normal cells beyond normal counterparts (i.e. HSPCs) when rigorous data were available. Furthermore, we provide an updated overview of the current AML CAR T-cell therapies worldwide, offering a state-of-art review of an unmet and urgent need in this emerging field.

## AML targets for CAR T-cell therapy

No ideal CAR T-cell therapy target (i.e. a cell surface molecule exclusively expressed on AML cells including leukemic stem cells and absent in any normal tissues) for AML patients has been identified, yet. In current clinical practice, CD33 and CD123 represent the most commonly utilized molecules for CAR T-cell engineering. This type of targets includes normal proteins overexpressed in AML and thus, they are classified as tumor-associated antigens (TAA) and are generally expressed in normal tissues as well, raising concerns of on-target off-tumor toxicity. Targeting myeloid antigens is more challenging than CD19 for lymphoid malignancies as myeloid targets are generally expressed on normal myeloid progenitor cells as well as HSCs and thus, targeting this type of tumor-associated antigens may lead to significant myelotoxicity. Over-expressed antigens can often be further classified into myeloid (CD123, CLL-1, CD33, CD64, CD116, CD13, CD93) or re-purposed lymphoid (CD19, CD7, CD70) markers. Neoantigens in AML are few and mainly intracellularly located. This is believed to be due to the fact that AML bears fewer albeit frequently recurring genetic mutations compared to solid tumors (7). Potentially cancer-specific targets are those derived from alternative mRNA splicing such as CD44v6 or molecules that are up-regulated or subcellularly re-localized upon cellular stress (PR1 peptide, GRP78) or chemical induction (CD38, FRβ).

We reviewed all these types of AML targets that have been proposed as CAR T-cell targets in AML either in proof-of-principle strategies that we call pre-clinical or more rigorous strategies actively being used in clinical trials. We specifically looked at the expression profile of these molecules in both leukemic and normal cells based on extensive review of current literature and mining large datasets (**Table 1**). We carefully reviewed all available clinical trial data published on clinicaltrials.gov for CAR T-cell therapy used in refectory/relapsed (r/r) AML patients. We provide an overview of all clinical trials showing enrollment status, type of adoptive cellular therapy (T-cell, γδT-cell, or NK cell), phase classification, and recruiting sample size for each AML CAR target (**Table 2**) as of October 7^th^ 2022.

**Table 1:** Characteristics of AML target antigens proposed for CAR T-cell therapy: .

AML Antigen	Common Alias(es)	Clinical Trial Status	Associated Effector Functions	Normal Cellular Expression Profile	AML Patient Prevalence with Antigen Expression (%)	% bulk cells OR % LSC Antigen Expression	CAR CombinationalStrategies	Reference(s)
**CD123**	IL-3RA	Phase I/II Phase II/III (with CLL-1)	Production and differentiation of HPCs into myeloid and lymphoid lineage-restricted cells	HPCs, monocytes, megakaryocytes, plasmacytoid dendritic cells, and B-lymphocytes	45% - 95% (8) (9–11)	LSC: 95.3% (12)	CD33 CLL1	(13, 14)
**CLL-1**	CLEC12A, DCAL-2, MICL, CD371	Phase I/II Phase II/III (with CD123)	Cell adhesion, cell-cell signaling, glycoprotein turnover, inflammation, immune response; negative regulator of granulocyte and monocyte function	HSCs (2.5%), HPCs, myeloid progenitors, mature myelocytes (15)	85% - 92% (16)	Bulk cells: 77.5% – 92% LSC: 45.1% (12)	CD123 CD33	(17)
**CD19**	B-lymphocyte surface antigen B4	Phase II/III	B-cell differentiation	B- lymphocytes	3.2% (AML, n = 527) with 66% (mixed-phenotype, n = 3) (18)	Bulk cells: 5% - 34% (AML) (18)	–	(18)
**CD7**	TP41	Phase I/II	T-cell/B-cell interaction during early lymphoid development, mature T-cell interactions	Thymocytes, NK cell precursors, T-lymphocytes	30% (n = 20) (19)	Bulk cells: 95.6% (n = 1) (20) Blast: 45.1% (21)	–	(19, 20)
**CD33**	SIGLEC3	Phase I/II	Negative regulator of cytokine production and monocyte activation	HPCs, myeloid progenitors, mature myelocytes	85% - 90% (8)	LSC: 88.7% (12)	CD123 CLL1	(22)
**CD38**	ADPRC1	Phase I/II	Synthesis and hydrolyzes cyclic adenosine 5’-diphosphate-ribose (intracellular calcium ion mobilizer)	Myeloid progenitors, T-lymphocytes (activated), B-lymphocytes (activated), monocytes, NK cells	37.3% (M1), 46.9% (M2), 5.1% (M3), 38.1 (M4), 54.6% (M5), 45.5% (M6) (n = 304) (23)	Bulk cells: 95% (n =6) (24) Blast: 83% (n=93) (25) Blast: 78% (n = 304) (23)	–	(24, 26)
**CD44v6**	HCAM, Pgp-1, HUTCH-1, LHR	Phase I/II	Cell-cell interactions, cell adhesion, migration; lymphocyte activation, recirculation & homing, hematopoiesis, metastasis	Monocytes	64% (n = 25) (27)	Not Reported	–	(27, 28)
**CD70**	TNFSF7	Phase I/II	Proliferation of stimulated T-cells, enhance cytotoxic T-cell generation, T-cell activation	B- lymphocytes (activated), T-lymphocytes (activated)	86% (n = 30) (5)	LSC: >75% (5)	–	(29, 30)
**FLT3**	CD135, FLK2	Phase I/II	Apoptosis, proliferation, and differentiation of hematopoietic stem cells in bone marrow	HPCs, GMPs, B-lymphocytes, Dendritic cells, NK cells	70% - 100% (31) Mutated 27% (n = 854) (32)	Bulk cells: 5% -10% (ITD mutation), 2% - 5% (TKD missense mutation) (33)	–	(34)
**Siglec-6**	CD327, CD33L, OBBP1	Phase I/II	Putative adhesion molecule mediates sialic-acid dependent binding to cells	Mast cells, granulocytes, syncytiotrophoblasts, cytotrophoblasts	100% (n = 20 using biotinylated anti-Siglec-6 mAb) (35)	Bulk cells: 76.9% (35)	–	(35)
**NKG2DL**	CD314, KLR	Phase I/II	Activator of NK and T cells; Immune recognition of cellular stress	NK cells (mature), NKT (mature), γδ T lymphocytes (mature)	75% - 80% (36, 37)	Bulk cells: 67% to 100% MICA/B and/or ULBP2-3 (36, 37)	–	(38, 39)
**WT1**	WIT-2, WAGR, AWT1	Phase I/II	Organ development, differentiation, proliferation, and apoptosis	Mesodermal tissues (HSCs, myoepithelial progenitors, renal podocytes, ovary and testis)	88.3% (n = 60) (40) 70% - 90% (41)	Not Reported	–	(42, 43)
**Lewis Y**	CD174, FUT3	Phase I	Embryogenesis, tissue differentiation, tumor metastasis, inflammation, and bacterial adhesion	Granulocytes, synctiotrophoblasts	52% (n = 29) (44) 46% (n = 52) (45)	Not Reported	–	(46)
**ADGRE2**	EMR2	Phase I	Promotes cell-cell adhesion through interaction with chondroitin sulfate chains, promotes granulocyte chemotaxis, degranulation & adhesion	Monocytes, macrophages, Kupffer cells, granulocytes, neutrophils	100% (n = 109) (47) 93% (n = 30) (5)	Bulk cells: 87.8% (47) LSC: >75% (5)	–	(48)
**ILT3**	LILRB4, CD85k, LIR5	Phase I	Inhibitor of MHC class 1 immune activation	Monocytes, Macrophages, Dendritic Cells	100% (M5) (n = 17) (49) 97% (M4, n = 33), 100% (M5, n = 26) (50)	Bulk cells: 99% (Monocytic AML) (49) Bulk cells: ~75% (M4), ~100% (M5) (50)	–	(49)
**B7-H3**	CD276	N/A	May regulate T-cell mediated immune response	Trophoblasts	27% (n =111) (51)	Bulk cells: 57.2% (n = 8, mean bone marrow) (52)	CD70	(52–54)
**FRβ**	FBP	–	Folate receptor and transporter; DNA synthesis, methylation, and repair	Myeloid-lineage hematopoietic cells	68% (n=78) (55)	Not Reported	–	(56, 57)
**PR1 peptide**	VLQELNVTV	–	HLA-A2 peptide from leukemia-associated proteinase 3 and neutrophil elastase presented to cytotoxic T cells	N/A	Elevated (n = 9) (58)	Not Reported	–	(59)
**CD64**	Fc-gamma receptor 1	–	Innate and adaptive immune response to Fc region of immunoglobulin gamma	Myeloid cells, monocytes, macrophages, endothelial cells (cytokine induction), Neurons (cytokine induction)	46.8% (n = 64) (60)	Bulk cells: Variable (60)	–	(61)
**CD116**	CD116/CD131 complex GM-CSF Receptor (mutated)	–	Production, differentiation, and function of granulocytes and macrophages	GMPs, Granulocytes, Macrophages	CD116 overexpressed 63% - 78% (62) 83% (n=29) (63)	Bulk cells: 1% - 98.1% (63)	–	(63)
**c-KIT**	SCFR, PBT, CD117	–	Regulation of cell survival, proliferation, hematopoiesis, stem cell maintenance, gametogenesis, mast cell development, migration	Enhanced in granulocytes, melanocytes, inhibitory neurons, monocytes, breast glandular cells	64.9% (n = 75) (64) 70%-100% (65)	Bulk cells: 70% (65)	–	(65)
**GRP78**	HSPA5, BIP	–	May facilitate viral attachment and entry to host cells	Upregulated during cellular stress	>50% (n = 14), overexpressed in TARGET, TCGA microarray data, & MILE study (66),	Not Reported	–	(66)
**IL1RAP**	IL1R3	–	Component of interleukin 1 receptor complex which induces synthesis of acute phase and proinflammatory proteins during infection, tissue damage, or stress	Hepatocytes, placenta, monocytes, peripheral mononuclear cells	79% (n = 29) (67)	Not Reported	–	(68)
**TIM-3**	HAVCR2	–	Regulates macrophage activation, inhibits Th1-mediated auto- and alloimmune responses, promotes immunological tolerance	T lymphocytes (mature), Treg cells, macrophages, NK cells, dendritic cells, mast cells	[Vast majority] (69)	Bulk cells: 87.3% LSC: 78.5% (12)	CD13	(70)
**CD13**	ANPEP, Alanyl Aminopeptidase LAP1, PEPN, GP150, HAPN	–	Digestion of peptides, metabolisms of regulatory peptides, promote angiogenesis, tumor growth, metastasis	Enterocytes, hepatocytes, exocrine glandular cells, proximal tubule cells, prostate glandular cells, decidual cells	44% (n = 12) (71)	Not Reported	TIM-3	(72, 73)
**CD93**	C1QR1	–	Phagocytosis of apoptotic cells	Neutrophils, monocytes, plasmacytoid dendritic cells, myeloid dendritic cells, PBMCs	56.7% (74)	Not Reported	–	(75)

Summary of AML antigens currently under preclinical and clinical investigation by clinical trial status, associated effector function, normal cellular expression profile, AML patient prevalence of antigen expression, % bulk cells OR % LSC antigen expression (at diagnosis), and CAR combinational strategies. Please note that % of patient positivity may not necessarily reflect antigen expression levels.

**Table 2:** Clinical Trials with CAR T-cells in AML patients as of October 7^th^ 2022.

Trial #	NCT#	Enrollment Status	Adoptive Cell Therapy	Phase	Recruiting Sample Size (# of patients)	Target(s)
1	NCT04796441	Recruiting	γδT	NA	20	CD19
2	NCT05388305	Recruiting	γδT	NA	30	CD123
3	NCT04766840	Not yet recruiting	T	1	9	IM73
4	NCT05463640	Not yet recruiting	T	1	20	ADGRE2
5	NCT05467254	Not yet recruiting	T	1	20	CD33, CLL1
6	NCT05473221	Not yet recruiting	T	1	20	CD33
7	NCT05467202	Not yet recruiting	T	1	20	CLL1
8	NCT04803929	Recruiting	T	Early 1	25	ILT3
9	NCT03631576	Unknown	T	2,3	20	CD123, CLL1
10	NCT04010877	Recruiting	T	1, 2	10	CLL1 + CD123 and/or CD33
11	NCT04169022	Recruiting	T	NA	50	IL1RAP
12	NCT04219163	Recruiting	T	1	18	CLL1
13	NCT05023707	Recruiting	T	1,2	5	FLT3
14	NCT05252572	Recruiting	T	Early 1	36	CLL1
15	NCT05239689	Recruiting	T	Early 1	36	CD38
16	NCT05488132	Recruiting	T	1,2	20	Siglec-6
17	NCT03796390	Unknown	T	1	15	CD123
18	NCT04257175	Recruiting	T	2,3	10	CD19
19	NCT03585517	Unknown	T	1	15	CD123
20	NCT03222674	Unknown	T	1,2	10	Muc1, CLL1, CD33, CD38, CD56, CD123
21	NCT05248685	Recruiting	T	1	20	CD33, CLL1
22	NCT04658004	Not yet recruiting	T	Early 1	36	NKG2D
23	NCT04884984	Recruiting	T	1,2	20	CLL1
24	NCT04599543	Not yet recruiting	T	Early 1	36	CD123
25	NCT03473457	Terminated	T	NA	2	Muc1, CD34, CD133, CD117, CD123, CD56, CD33, CD38
26	NCT02944162	Unknown	NK	1, 2	10	CD33
27	NCT05266950	Recruiting	T	1	7	CI-135
28	NCT03114670	Unknown	T	1	20	CD123
29	NCT05445011	Recruiting	T	1	12	FLT3
30	NCT05215015	Recruiting	NK	Early 1	18	CD33, CLL1
31	NCT04623944	Recruiting	NK	1	90	NKG2D
32	NCT04097301	Terminated	T	1, 2	8	CD44v6
33	NCT05445765	Not yet recruiting	T	1	10	CD33
34	NCT03126864	Terminated	T	1	11	CD33
35	NCT03904069	Not yet recruiting	T	1	40	FLT3
36	NCT04835519	Recruiting	T	1,2	25	CD33
37	NCT04014881	Unknown	T	1	50	CD123
38	NCT04692948	Recruiting	T	NA	5	CD276
39	NCT03556982	Unknown	T	1,2	10	CD123
40	NCT04351022	Recruiting	T	1,2	20	CD38
41	NCT04318678	Recruiting	T	1	32	CD123
42	NCT05017883	Recruiting	T	NA	5	FLT3
43	NCT05432401	Recruiting	T	Early 1	18	FLT3
44	NCT04789408	Recruiting	T	1	40	CLL1
45	NCT05247957	Recruiting	NK	1	9	NKG2D
46	NCT03971799	Recruiting	T	1,2	37	CD33
47	NCT02623582	Terminated	T	Early 1	7	CD123
48	NCT04272125	Recruiting	T	1,2	40	CD123
49	NCT03612739	Withdrawn	T	1	0	NKR-2
50	NCT04923919	Recruiting	T	Early 1	100	CLL1
51	NCT02799680	Unknown	T	1	12	CD33
52	NCT04265963	Recruiting	T	1,2	45	CD123
53	NCT05008575	Recruiting	NK	1	27	CD33
54	NCT01864902	Unknown	T	1,2	10	CD33
55	NCT05105152	Recruiting	T	1	18	CD33
56	NCT05016063	Not yet recruiting	T	Early 1	32	CLL1
57	NCT02203825	Completed	T	1	12	NKG2D
58	NCT03896854	Recruiting	T	1,2	15	CD19
59	NCT03291444	Recruiting	T	1	30	Muc1, CD34, CD123, CD117, CD56, CD38, CD33
60	NCT02159495	Active, not recruiting	T	1	31	CD123
61	NCT05092451	Not yet recruiting	NK	1, 2	94	CD70
62	NCT03885076	Unknown	γδT	N/A	20	CD33
63	NCT05457010	Not yet recruiting	T	1	24	CD123
64	NCT04106076	Withdrawn	T	1	0	CD123
65	NCT03190278	Recruiting	T	1	65	CD123
66	NCT04662294	Recruiting	T	Early 1	108	CD70
67	NCT03927261	Recruiting	T	1	88	CD33
68	NCT05442580	Not yet recruiting	T	1	36	CD38
69	NCT04230265	Recruiting	T	1	45	CD123
70	NCT04762485	Recruiting	T	1,2	20	CD7
71	NCT04033302	Recruiting	T	1,2	30	CD7
72	NCT05513612	Recruiting	T	1	20	CD19, BCMA, CD123, CD7
73	NCT01716364	Not yet recruiting	T	1	6	Lewis Y
74	NCT05454241	Not yet recruiting	T	2	22	CD7
75	NCT03672851	Terminated	T	1	2	CD123
76	NCT03018405	Unknown	T	1,2	146	NKR-2
77	NCT03766126	Active, not recruiting	T	1	12	CD123
78	NCT04678336	Recruiting	T	1	12	CD123
79	NCT02742727	Unknown	NK	1,2	10	CD7
80	NCT04167696	Recruiting	T	1	27	NKG2D
81	NCT05377827	Not yet recruiting	T	1	48	CD7
82	NCT04156256	Unknown	T	Early 1	20	CD33, CD123
83	NCT03795779	Recruiting	T	Early 1	20	CD33, CLL1
84	NCT05518357	Recruiting	T	Phase 1	15	ILT3

Summary of CAR T-cell AML therapies published on clinicaltrials.gov by enrollment status, phase, recruitment size, and target selection as per October 7^th^ 2022.

## Targets under clinical investigation for CAR T-cell therapy of AML

### CD123

CD123, also known as IL-3Rα, is a membrane protein that is highly expressed in ~80% of AML patients (although varied levels of expression across patients) and is closely related to AML patient prognosis. CD123 overexpression has been identified on leukemic stem cells (LSCs) and AML blast cells with minimal expression on normal HSCs. CD123 expression has been reported on 95% of LSCs (12). In addition to AML, CD123 has been implicated in other myeloid malignancies such as Chronic Myelomonocytic Leukemia (CMML), Myelodysplastic Syndrome (MDS), Myeloproliferative Neoplasms (MPN), and Chronic Myeloid Leukemia (CML), and blastic plasmacytoid dendritic cell neoplasm (BPDCN) (76). Stimulation of CD123 with IL-3 functions as an important promoter of proliferation and differentiation of hematopoietic progenitor cells (HPCs) into both myeloid-derived and lymphoid-restricted lineages. CD123 is expressed on IL-3-responsive cells such as HPCs, monocytes, megakaryocytes, plasmacytoid dendritic cells, and B-lymphocytes. Several studies have targeted CD123 in AML including recombinant fusion proteins, anti-CD123 neutralizing monoclonal antibodies, and CAR T-cell therapies. In the field of CAR T-cell therapy for AML, CD123 is currently the most utilized target with over 20 clinical trials either completed or ongoing. In one study by Khawanky et al., third-generation anti-CD123 CAR T-cells showed significant anti-AML activity when combined with DNA methyltransferase inhibitor, AZA (13). Currently, several anti-CD123 CAR T-cell therapies are in phase I and phase I/II clinical trials including combinatorial targeting with anti-CLL-1 CAR T-cell therapy in active phase III. As of this writing, there are two terminated anti-CD123 CAR T-cell trials. Seven r/r AML patients were enrolled in phase I clinical trial at the University of Pennsylvania, United States by Gill et al. in which patients were given “biodegradable” T-cells transiently expressing anti-CD123 CAR (77). These T-cells were *ex vivo* electroporated with anti-CD123 mRNA to express anti-CD123 CAR. Despite no treatment-related death nor any clinical hematologic suppression, there was no reduction in CD123 expressing cells in the bone marrow, and all patients who were treated had disease progression before day 28.

### CLL-1

CLL-1 or CLEC12A is a C-type lectin-like receptor that has a critical role in cell adhesion, cell-to-cell signaling, inflammation, and serves as a negative regulator of granulocyte and monocyte function. CLL-1 is a commonly studied marker of AML due to its relatively high expression on both leukemic stem cells (approximately 45%) and leukemic blasts (between 77.5% to 92%) with a high proportion of AML patients (> 85%) expressing CLL-1. Clinical measurement of CLL-1 has been considered a reliable marker for minimal residual disease and risk stratification/prognosis of AML patients (78). In a phase I clinical trial at Nankai University, China by Jin et al., ten r/r AML patients treated with anti-CLL-1 CAR T-cells demonstrated a 70% complete remission or complete remission with incomplete hematological recovery (CR/CRi) (17). In addition to CD123, anti-CLL-1 CAR T-cells has been combined with anti-CD33 CAR T-cells, however clinical results have yet to be published.

### CD19

CD19, also known as B-lymphocyte surface antigen B4, is a marker of B-cell differentiation and has restricted expression on B-lymphocytes. CD19 is critical for regulating intrinsic B-cell activation thresholds *via* B-cell receptor-dependent and independent signaling pathways (79). CAR T-cells targeting CD-19 have been well studied in the context of ALL, but less so for AML. Aberrant expression of CD19 has been identified in AML subtypes with the t(8:21) translocation and can often present in cases without this genetic aberration (80).

In a study of 527 AML patients, the overall CD19 expression was 3.2%, with 17 out of 527 AML patients expressing CD19. CD19 was expressed in 2 out of 3 mixed-phenotype acute leukemia patients (66%) (18). Bulk cells have been shown to express anywhere between 5% to 34% of CD19 in these patients. While strategies of CAR-T cell targeting of CD19 for AML have been limited, one study for CD19+ AML patients has made it to phase 2 & 3 clinical trial and is actively recruiting at the Sheba Medical Center, Israel.

### CD7

CD7 is a glycoprotein that is functionally essential for T-cell and T-cell/B-cell interactions during early lymphoid development. Expression of CD7 is common on thymocytes, NK cell precursor cells, and T-lymphocytes. In one study CD7 expression was found in approximately 30% of AML patients (n = 30) with high expression linked with more aggressive disease status and resistance to traditional therapy (19). Elhadi et al. showed that CD7 positivity was detected on 45% of AML Sudanese patients (n = 51) with relatively minimal expression compared to other markers; the mean positivity among all cases reported was 27.4% (21). While positive for CD7, minimal expression of CD7 on AML blast cells on patients limits their potential enrollment in clinical trials. In a 2019 study, Gomes-Silva et al. demonstrated that a CD7-directed CAR in CD7 gene-knockout T cells could effectively eliminate CD7+ leukemic cells in mice while protecting against significant systemic myeloablation (19). Efforts to genetically remove endogenous CD7 for an “off-the-shelf” anti-CD7 CAR-T cell therapy have been made to limit effector T cell fratricide (81). In a phase I clinical trial at Soochow University, China, one patient with a complex karyotype, *TP53* deletion, *FLT3-ITD* mutation, and *SKAP2-RUNKX1* fusion gene, expressed blasts with 95.6% CD7 positivity (20). After seventeen days post anti-CD7 CAR T-cell infusion, the patient achieved morphologic leukemia-free state. Despite a consequent grade 3 cytokine release syndrome (CRS), the patient did not experience organ failure or immune effector cell-associated neurotoxicity syndrome (ICANS) and received allo-HSC transplantation with CR without MRD.

### CD33

Also known as Siglec3, CD33 is a surface marker that binds and becomes stimulated by sialic acid residues such as glycoproteins or glycolipids. CD33 serves as a negative regulator of cytokine production and monocytic activation commonly expressed on HPCs, myeloid progenitors, and mature myelocytes. Present in over 85% of AML patients, CD33 has been shown to have a high expression on LSCs (88.7%) (8, 12). CD33 lack of expression on HSCs has gained importance as a potential tumor-associated antigen marker for targeting in AML. Several CAR T-cell therapies against CD33 have made it to clinical trial phases 1 and 2 with seven actively recruiting and different cell type modalities being used such as CAR T-cell, CAR γδT-cell, and CAR NK-cell. In the single terminated CD-33 CAR T-cell clinical trial at M.D. Anderson Cancer Center, United States, three adult r/r AML patients received anti-CD33 CAR T-cells at a dose level (0.3 x 10^6 cells/kg) (22). Two of the three patients developed toxicities including grade 3 tumor lysis syndrome, acute kidney injury, grade 2 mucositis, grade 1 tachycardia, and grade 2 intermittent orthostatic hypotension with increases in bilirubin and ALT/AST levels. Despite CAR T-cell detection in peripheral blood, all three patients died from AML disease progression by 31 days post CAR T-cell infusion. No dose-limiting toxicity was observed.

### CD38

CD38 a surface glycoprotein involved in cell adhesion, migration, and signal transduction. Present in myeloid progenitors, B- and T-lymphocytes, monocytes, and NK cells, CD38 plays an important role in intracellular calcium ion mobilization. Pre-exposing leukemic cells with all-trans-retinoic acid (ATRA) has been shown to increase CD38 expression on AML cells and enhance the cytotoxic effects of anti-CD38 CAR T-cells in AML cell lines (26). Within the AML patient population, between 5.1% (lowest, M3) to 54.6% (highest, M5) have expression of CD38 with blast expression approximately 78% (23). Anti-CD38 CAR T-cells are currently being studied in phase I/II clinical trial. In phase I/II clinical study at Soochow University, China, by Cui et al., six patients exhibited a median CD38 blast expression of 95%. Two-thrids (66.7%) of patients in this study achieved CR/CRi within four weeks following anti-CD38 CAR T-cell infusion (24). Of the adverse events reported, 5 patients experienced CRS grade 1-2 and one patient with CRS grade 3, no patients with ICANS, and all patients with neutropenia and thrombocytopenia (cell and platelet counts less than 500/μl and 10,000/μl respectively). Interestingly, of the one of two patients who relapsed, one patient achieved a CR after second treatment with anti-CD38 CAR T-cells.

### CD44v6

The hyaluronate receptor CD44 is a surface glycoprotein that is highly implicated in specific processes such as leukocyte activation and malignant transformation. As an adhesive receptor, CD44 mediates cell-to-cell and cell-to-extracellular matrix interactions by binding to its main ligand, hyaluronic acid, a glycosaminoglycan highly concentrated in the bone marrow niche (82). CD44 is a useful marker for prospective identification of cancer stem cells (83). While the normal isoform of CD44 is expressed ubiquitously in both normal adult and fetal tissue, certain splice-isoforms of CD44 are relatively tumor-restricted (84, 85). In particular, CD44v6 is the most well-known splice variant of CD44 and has been associated with a poorer prognosis of AML (86). Abnormal CD44 splicing and expression has been demonstrated in other hematological and non-hematological malignancies such as NHL, myeloma, gastric mucosa, colon, and pancreatic cancers (87). As an AML-targeted therapy, CD44v6 is a promising candidate due to its lack of expression on HSCs and minimal expression on normal cells (88). CD44v6 shows 64% expression in AML patients (n = 25) (27). Monoclonal antibodies against CD44 have been shown to interfere with human leukemia initiation in immunocompromised mice (89) and second-generation anti-CD44v6 CAR T-cells have some efficacy *in vivo* and *in vitro* mouse models (28). While an anti-CD44v6 CAR T-cell phase I/II clinical trial by AGS Biologics S.p.A. began in August 2019, it was terminated due to inability to close study in a clinically relevant time frame.

### CD70

CD70 is a member of the TNF receptor family whose expression is present on antigen-presenting cells upon maturation. Interaction with CD27 stimulates CD70 to promote effector CD8+ T cell response, influence polarization of CD4+ T-cells, and suppress effector Th17 function (90). Within AML pathogenesis, CD70 may have some influence in activating stem cell expression programs including the Wnt signaling pathway and promoting cellular division/proliferation (91). Over 85% of AML primary patient samples have been found with CD70 expression over 75% of their LSCs being positive for CD70 (5). Sauer et al., investigated several anti-CD70 CAR constructs with different hinges (IM, LF) and costimulatory molecules (CD27, CD28, and 4-1BB) and they report significant *in vitro* and *in-vivo* tumor-killing with LF-28z and CD27z CAR T-cells in two AML xenograft models without HSC colony assay toxicity (29). Azactidine (AZA) has been shown to increase expression of CD70 through hypomethylation of the CD70 promoter (92). Enhanced CD70 expression with anti-CD70 CAR T-cells have shown promising preclinical results *in vitro* and *in vivo* models. A non-cleavable hinge in the anti-CD70 CAR construct demonstrates enhanced binding, expansion, and potent *in vivo* activity and synergy with AZA (30). There is currently one anti-CD70 CAR T-cell clinical trial at Zhejiang University, China recruiting in early phase I and one not yet recruiting anti-CD70 NK-cell phase I/II clinical trial at M.D. Anderson Cancer Center, United States.

### FLT3

FLT3 (Fms-like tyrosine kinase 3) is a well-known receptor ligand for stem cell maintenance and differentiation. FLT3 is expressed in hematopoietic progenitor cells, granulocyte-myeloid progenitor cells, B-cells, dendritic cells, and NK cells. Mutations in the FLT3 receptor can lead to abnormal myeloid development. FLT3 mutations can be classified into either internal tandem duplicates (ITD) (representing approximately 25% of AML patients) and point mutations in the tyrosine kinase domain (TKD) (approximately 5% of AML patients) (93). Between 70% of 100% of AML patients have increased expression of FLT3 with ITD mutations having increased relapse rates, reduced disease-free survival, and decreased long-term survival (31, 94). Anti-FLT3 CAR T-cells have been shown to kill FLT3+ cell lines and AML patient bone marrow mononuclear cells *in vitro* with more potency in FLT3-ITD cells with an effector to target (E:T) ratio of as low as 1:8 (34). There are four recruiting clinical trials for anti-FLT3 CAR T-cells, but no results have been published yet.

### Siglec-6

Siglec-6 is a putative cell adhesion molecule that binds sialic-acid on mast cells, granulocytes, syncytiotrophoblasts, and cytotrophoblasts. Siglec-6 prevents primary mast cell activation and may be relevant for mast cell activity in colorectal cancer (95). Siglec-6 has mild expression in mucosal lymphoid cells and high expression in synctiotrophoblasts of the placenta. Using flow cytometry biotinylated anti-Siglec-6 monoclonal antibodies, Jetani et al. report 100% (n = 20) AML patients expressing Siglec-6. Within this group, over 75% of bulk AML cells expressed Siglec-6. Anti-Siglec-6 CAR T-cells have shown promising *in vivo* and *in vitro* cell killing activity with E:T ratios ranging from 10:1 to 1:4 against AML cell lines including U937, MV4;11, and MOLM-13 (35). Development of CAR T-cell for Siglec-6 is currently recruiting for phase I/II clinical trial at Xuzhou Medical University, China.

### NKG2DL

Natural killer group 2, member D ligand (NKG2DL) is an activating receptor of NK cells and T lymphocytes for immune recognition of abnormal cells (i.e. tumor cells). Targeting the NKG2D/NKG2D ligand axis is a new potential avenue for both biomolecular and adoptive cellular therapies for AML. NKG2D interacts with MHC class I related molecules (upregulated during cellular stress), which in humans, consist of either MHC class I related chain (MIC) family (MICA, MICB) or UL16-binding protein (ULBP) family (ULBP1-6) (96). Stimulating NKG2D can activate immune cells to recognize the NKG2D ligands present on AML cells. Resistance to chemotherapy and immune recognition has been associated with downregulation of NKG2D. According to a 2012 study by Hilpert et al., the leukemic cells of 70% of AML patients (n = 104) were positive for at least one NKG2D ligand (36). Within 15% of total cases, AML cells expressed four or five different NKG2D ligands (36). There is only one completed clinical trial with anti-NKG2DL CAR cells. In a first in-human AML Phase 1 study at Dana-Farber Cancer Institute and National Heart, Lung, and Blood Institute (NHLBI), Baumeister et al. were to achieve appropriate safety or feasibility levels, but unfortunately unable to elicit an efficient clinical response (38). Driouk et al. demonstrate increased NKG2D CAR T-cell efficacy against AML cells *in vitro* upon pharmacologic NKG2D-ligand upregulation with HDAC inhibitors (39). Several other clinical trials are currently recruiting for both CAR T-cells and CAR NK-cells against NKG2D ligands.

### WT1

Wilms Tumor 1 (WT1) has been extensively studied over several decades in its deterministic role of AML pathophysiology. Either overexpression or mutation of WT1 leading to cancer has led to its interesting dual-role as both tumor suppressor and oncogene, depending on the context (97). WT1 signaling is essential to organ development of mesodermal tissue including HSCs, myoepithelial progenitor cells, and kidney podocytes. Between 70% to 90% of AML patients have elevated expression of WT1 on AML cells (41). One phase I/II clinical trial at University College, London, has been completed with anti-WT1 CAR T-cells demonstrating a Relapse-Free Survival (RFS) of 100% after 44 months following infusion in 12 patients compared to 54% RFS in 88 patients without CAR T-cell therapy after allogenic HSC transplantation (42).

### Lewis Y

Lewis Y is a surface glycoprotein present on granulocytes and synctiotrophblasts during embryogenesis. Lewis Y antigen is a difucosylated oligosaccharide and its presence is highly correlated with the pathological staging and prognosis of epithelial ovarian cancer (98). Lewis Y is suggested to promote tumor survival, invasion, and metastasis (99). Within AML patients, reports indicate that up to approximately 50% of patients have expression of Lewis Y on leukemic cells. In the first AML CAR T-cell therapy trial a phase 1 clinical study at Peter MacCallum Cancer Center, Australia, with anti-Lewis Y CAR T-cells for r/r AML patients, Ritchie et al. demonstrate mild feasibility and safety for targeting Lewis Y and CAR T cell persistence up to 10 months in proven sites of disease (leukemia cutis and bone marrow) (46). Although limited efficacy was observed overall, this was an important study to assess the potential of CAR-T therapy in AML patients and its use with limited hematological suppression. Neeson et al. engineered anti-Lewis Y CAR T-cells using autologous T-cells based on the Lewis-Y ex-vivo transduction and expansion protocol, showing a cytolytic response against Lewis Y positive AML cells *via* production of interferon-γ (100).

### ADGRE2

ADGRE2 or EMR2 is a cell adhesion protein that interacts with chondroitin sulfate chains and promotes granulocyte chemotaxis, and degranulation. ADGRE2 expression is present on granulocytes, monocytes, macrophages, Kupffer cells, and neutrophils. Expression of ADGRE2 in AML was first identified in a set of four candidate targeting proteins by Perna et al. with 93% of patients (n = 30) having greater than 75% protein expression on LSCs (5). Haubner et al. recently created an anti-ADGRE2 CAR T-cell model with a chimeric-costimulatory receptor against CLEC12A (48). In this, both ADGRE2 and CLEC12A must be present in the tumor for the CAR T-cell to be activated. This gating strategy is proposed to maintain T-cell cytotoxicity and tumor-killing efficacy within the tumor microenvironment and lead to long-term potentiation of the infiltrating CAR T-cells. Using NSG *in-vivo* xenografts of engineered low-expressing ADGRE2 MOLM13 AML cells to model antigen escape, Haubner et al. showed ADGRE2 and CLEC12A had increased performance than alternative dual (OR-gated) ADGRE2 CLEC12A CAR T-cells with functional persistence >70 days. There is one clinical trial that has not yet begun recruiting at Zhejiang University, China.

### ILT3

ILT3 or LILRB4 is an inhibitor of MHC class-I immune activation and present on monocytes, macrophages, dendritic cells, endothelial cells, and osteoclasts. ILT proteins are structurally and functionally similar to the killer cell immunoglobulin-like receptors (KIR) expressed on NK cell and T-lymphocytes. Expression of ILT3 from antigen-presenting cells can induce anergy of CD4+ helper T-lymphocytes and differentiation of CD8+ T suppressor cells. ILT3 expression has been identified in both M4 and M5 AML subtypes (FAB classification) with M4 and M5 patients showing approximately 60% and 100% positivity (49, 50). While prevalence of ILT3 positivity is high in both M4 and M5, its expression intensity may change during leukemic disease progression (101, 102). Preclinical evaluation of an anti-ILT3 CAR T-cell model has demonstrated efficient effector function *in vitro* and *in vivo* against ILT3+ AML cells (MV4-11, PriAML-1, THP-1, PriAML-2, MOLM13, PriMonoctye) with an E:T ratio of 5:1 resulting in between approximately 50% to 90% specific cell lysis compared to only approximately 20% specific cell lysis in non-transduced control T-cells after 4 hours. Toxicity to CD34+ umbilical cord blood cells was also not present (49). An early phase 1 clinical trial at Zheijang Provincial People’s Hospital, China is currently recruiting patients with M4 and M5 AML, however results have yet to be published. Concurrently, an anti-LILBR4 chimeric synthetic T cell receptor and antigen receptor (STAR), which incorporates an antigen recognition domain and constant regions of a TCR, is actively recruiting in phase I clinical investigation at Hebei Yanda Ludopei Hospital, China and results have yet to be published.

## Targets under pre-clinical investigation for CAR T-cell therapy of AML

### B7-H3

B7-H3, also known as CD273, is a type I transmembrane protein that was initially first characterized as a T-lymphocyte stimulating protein. However, recent studies have suggested that B7-H3 serves as an inhibitor to T-cell cytotoxicity and promoter of immune evasion within cancer cells (103). B7-H3 is expressed on multiple cancer types including AML blasts (53). Several groups have begun to investigate B7-H3 as a potentially new immune checkpoint therapy with analogous function to the PD-1/PD-L1 immune tolerance axis (104–106). Within a cohort of AML patients, 27% had positivity for B7-H3 with highest expression in the M3 and M5 subtypes (51). Anti-B7-H3 CAR T-cells have shown promising results in preclinical *in vitro* and *in vivo* models (52, 53, 106). Lichtman et al. demonstrate an anti-B7-H3 CAR T-cell model that shows significant interferon-γ and IL-2 release with cytotoxicity against OCl-AML2, OCI-AML3, THP1, and U937 along with primary AML patient samples with E:T ratio of 1:5 after 48 hours (52). Yang et al. have shown efficacy with tandem anti-B7-H3 and anti-CD70 CAR T-cells *in vitro* and *in vivo* (54). At an E:T ratio of 8:1, the tandem CAR T-cells has approximately 50% and 60% specific cell killing compared to single CD70 CAR T-cell 35% and 40% specific cell killing for NHI-460 and A375 AML cell lines respectively.

### FRβ

Folate Receptor β (FRβ) is a receptor and transporter of folate which is a necessary molecule for DNA synthesis, methylation, and repair. FRβ represents a potentially useful marker for AML due to its relatively limited expression in myeloid-lineage hematopoietic cells. Approximately 70% of AML patients (n = 20) have shown to be positive for FRβ (55). While there is some concern for off-tumor toxicity against normal myeloid-lineage cells, anti-FRβ CAR T-cells have been reported to have mild cytotoxic efficacy *in vitro* against FRβ-expressing AML cell lines with minimal toxicity against normal CD34+ HSCs (56). To address the limited capacity of anti-FRβ CAR T-cells to kill low-expressing FRβ AML, addition of ATRA increasing FRβ expression enhances CAR T-cell tumor-killing. High affinity anti-FRβ AML CAR T-cells demonstrate >50% cell killing in C30-FRβ expressing, THP1, and MV411 AML cell lines with minimal killing in HL60 at E:T ratios 5:1 and 1:1 (57). FRβ CAR-T cells show limited toxicity against CD34- bone marrow cells in CFU assays.

### PR1 peptide

PR1 peptide is a 9 amino-acid long peptide derived from the myeloid proteinase 3 (PR3) and neutrophil elastase (NE). Stress-induced presentation of PR1 peptide in AML patients allows for cytotoxic T lymphocytes to bind *via* the MHC-HLA/TCR interaction. Targeting PR1 in AML is a promising choice due to both PR3 and NE high expression in myeloid malignancies including AML, MDS, and CML and very minimal expression in other tissues. Therapies such as a PR1 -peptide vaccine, anti-PR1/HLA-A2 antibody, and anti-PR1 CAR T-cells have been under development (58, 59, 107). Compared to a control CMV pp65 peptide HLA-expressing T2 cells, anti-PR1-peptide CAR T-cells (derived from the TCR-like 8F4 antibody) show increased % cell killing against PR1 peptide HLA-expressing T2 cells at E:T ratios between 4:1 to 0.5:1 (59). Additionally, anti-PR1 peptide CAR T-cells show rapid and effacing killing of AML *in vitro*.

### CD64

CD64 or Fc-gamma receptor 1 is a high-affinity receptor present in monocytes, macrophages, and neutrophils that mediates many immunological responses including phagocytosis, cytokine release, antigen presentation, and cellular cytotoxicity (108). CD64 is widely expressed in patient AML samples and serves as a potential target for CAR T-cell therapy. Thus far, reporting of CD64 expression in AML samples has remained variable with approximately 46.8% AML patients (n = 64) positive for CD64 (60). Blast % positive with CD64 is highest in the M5 subtype, but also present in M3, M2, and M4 with 80%, 58%, and 20% respectively. In both *in vitro* and *in vivo* models, anti-CD64 CAR T-cells demonstrate limited myeloid cell suppression and lack of toxicity to CD34+ HSCs (61). T-cells transduced with anti-CD64 CAR show approximately 80% specific cell lysis against U937 and THP-1 AML cell lines at E:T ratio 1:2 with control T-cells showing minimal killing effect (61).

### CD116

CD116 is the granulocyte-macrophage colony-stimulating factor (GM-CSF) receptor that complexes with CD131 to respond to GM-CSF which is a potent stimulator of myeloblast development and lymphoid production. GM-CSF is primarily located on neutrophils, eosinophils, monocytes, and macrophages. Mutated GM-CSF has been reported in juvenile myelomonocytic leukemia. CD116 is overexpressed in 63% to 78% of AML cases and Hasegawa et al. report a wide range of % blast positivity from 1% to 87.7% in AML patients (n = 29) with various subtypes (62). Hasegawa et al. use a mutated GM-CSF CAR at residue 21 to target CD116+ AML cells *in vitro*. These anti-CD116 CAR T-cells demonstrate durable cytotoxicity against AML cells (Kasumi-1, HL-60, ShinAML, THP-1, and MV4-11) preclinically with E:T ratios of 1:10 and 1:5 having reliable cell killing effects after 5 day co-culture with AML M4 and THP-1 M5 AML cell lines (63).

### c-kit

Stem cell factor (SCF) and its cognate receptor tyrosine kinase (c-kit), also known as CD117, is an important regulator of many hematological cellular processes including cell survival, proliferation, hematopoiesis, and stem cell maintenance. C-kit is highly expressed in HPCs and upregulated in most primary AML cells. Enhancement of c-kit can occur in granulocyte, melanocytes, inhibitory neurons, and monocytes. C-kit expression has been reported between 64% to 100% of AML patients with median bulk AML expression of 70% (64, 65). The generation and validation of anti-c-kit CAR T-cells *in vitro* and *in vivo* models show both CAR T-cell directed killing against AML cells and primary HPCs (65). Anti-c-kit CAR T-cells eliminated c-kit-mixed negative/low, c-kit intermediate, and c-kit high target cells at an E:T ratio of 1:10. While myeloablation is inevitable, Myburgh et al. propose this platform to be a potential bridging therapy for pre-transplant AML patients.

### GRP78

Glucose-regulated-protein 78 (GRP78) is a regulator of the unfolded protein response and may facilitate viral attachment and entry into host cells. GRP78 acts as a promising target for AML CAR T-cell therapy due to its normal residence in the endoplasmic reticulum in healthy cells and stress-induced transportation to the cell surface when a cell undergoes tumorigenesis. Over 50% of AML patients (n =14) have been reported to express GRP78 with gene overexpression also present in large databases such as TARGET, TCGA, and MILE (66). CAR T-cell against GRP78 show ability to recognize and kill GRP78 positive AML cells without toxicity to HPCs (66). Increased surface expression of GRP78 with dasatinib improves effector function of anti-GRP78 CAR T-cells. For single anti-GRP78 CAR expression, approximately 70% specific cell lysis occurred against MOLM13 AML cells compared to approximately 10% control T-cells at an E:T ratio of 0.5:1 (66).

### IL1RAP

Interleukin-1 receptor accessory protein (IL1RAP) is an indispensable type 1 receptor for IL-1 signaling. IL1RAP is widely expressed by multiple subtypes of AML and serves as a putative marker for leukemic stem cells (109). IL1RAP induces synthesis of the acute phase and proinflammatory proteins during infection, tissue damage, or stress. IL1RAP interacts with both FLT3 and c-KIT, two receptor tyrosine kinases with roles in AML pathogenesis. Approximately 80% of AML patients (n = 29) have expression of IL1RAP on blasts (67). Third-generation anti-IL1RAP CAR T-cells show significant cytotoxicity against primary AML at both time of diagnosis and relapse (68). Anti-IL1RAP CAR T-cells demonstrate significant tumor cell killing HL-60, MOLM-13, Mono-Mac-6, and KU812 AML cell lines.

### TIM-3

T-cell immunoglobulin mucin-3 (TIM-3) is a surface molecule expressed in interferon gamma (IFNγ)-producing T lymphocytes. TIM-3 has been identified as a marker of LSCs with lack of expression on normal CD34+CD38- HSCs but not CD34+CD38- LSCs in most types of AML except for acute promyelocytic leukemia (M3) (110). Kikushige et al. report that a vast majority of AML patients have increased expression of TIM-3 on bulk AML cells (41). Haubner et al. report bulk cell expression of TIM-3 to be 87.3% and LSC expression 78.5%. Anti-TIM-3 CAR T-cells exhibit effective AML cell killing and eradication of LSCs *in vitro* and *in vivo* models (70).

### CD13

CD13 or Alanyl Aminopeptidase is a plasma membrane peptidase enzyme that digests peptides on the surface of the small intestine. CD13 also has less clear function in proximal tubular cells and is thought to be involved in the metabolism of peptides that serve regulatory functions in a diverse array of cell types including macrophages, granulocytes, and neurons. Defects in CD13 have been an implicated cause of various leukemia and lymphoma. Khurram et al. report 44% of AML patients (n = 12) having aberrant expression of CD13 on blast cells along with patients with B-ALL and T-ALL (71). Due to the off-target expression of CD13 on gastrointestinal cells, He et al. deploy the use of a switchable CAR-compatible nanobody, Nb157, anti-CD13 CAR T-cell *in vitro* against THP-1 cells and CD13+ K562 cells (72). *In vivo* modeling demonstrated significant tumor reduction after day 21 switch injection and by day 52, all tumors were undetectable. Over 35% of HSCs and progenitors were detected in the BM of post-conditioned and HSC-transplanted mice following treatment of low dose (0.1 mg/kg) CAR T-cells.

### CD93

CD93 or C1QR1 is a cell-surface glycoprotein and type 1 membrane protein identified as a myeloid cell-specific marker. CD93 is thought to be involved in immunological clearance of apoptotic cells. Approximately 55% of AML patients have differential expression of CD93 and CD93 is associated with genomic rearrangements of the MLL (mixed lineage leukemia) gene (111). Richards et al. recently demonstrate a NOT-gated anti-CD93 CAR T-cells that can recognize CD123 and not engage thus limiting the cytotoxic effects of endothelial cell CAR T-cell recognition and killing (75).

**Figure 1:**
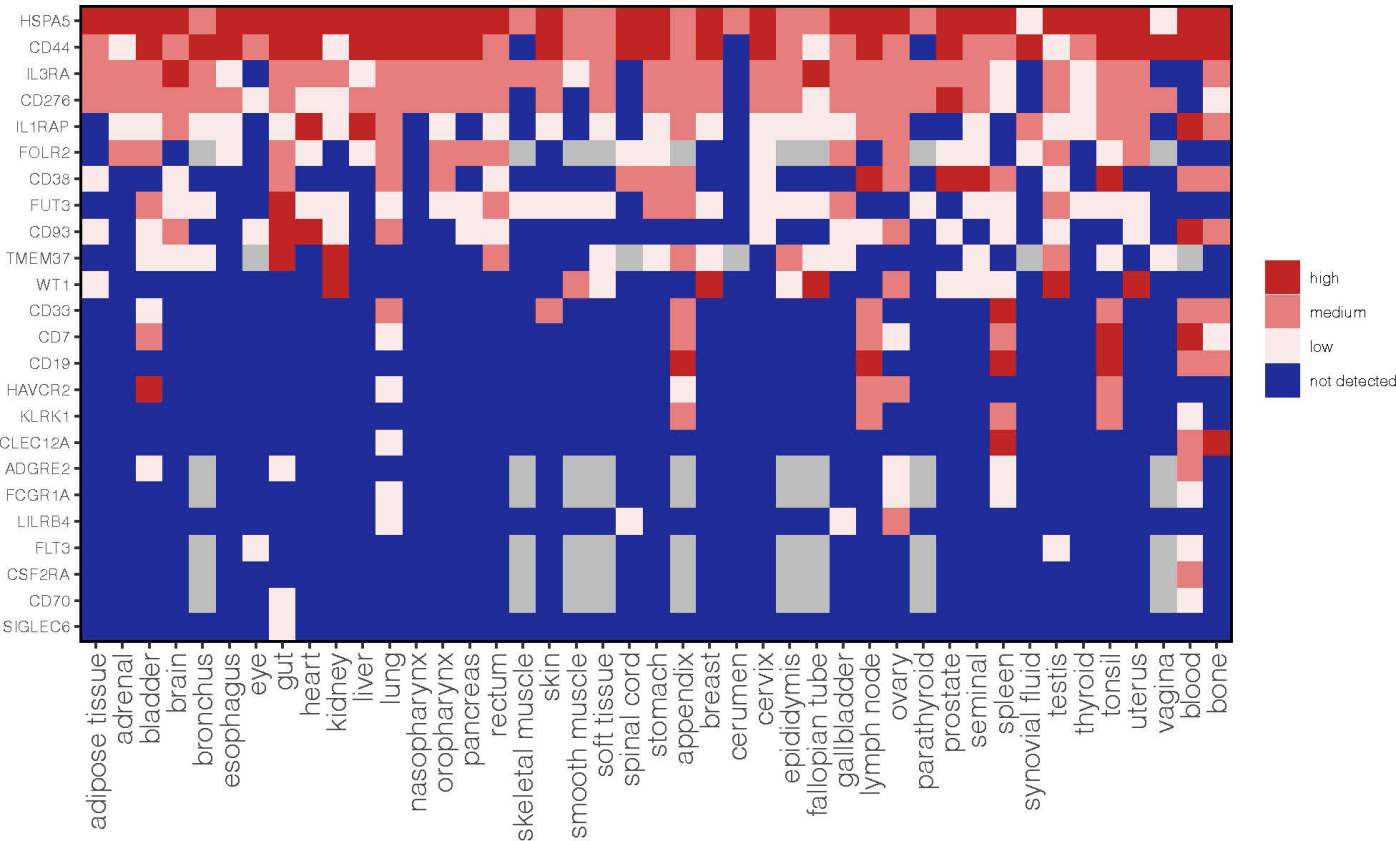
Expression profile of AML target antigens previously reported as AML CAR targets in normal tissues. CD44 refers to aggregate of CD44 isoforms rather than the CD44v6 CAR target. This is an updated figure from Perna F et al., Cancer Cell 2017 (**5**).

To provide a comprehensive analysis of these AML targets we also annotated the expression of each antigen in a large panel of normal tissues based on the integration of three publicly available protein databases such as the Human Protein Map, Human Protein Atlas and Proteomic Database (**Figure 1**). The methodology to merge these datasets and obtain an approximate cut-off for low, medium and high expression levels was previously described in Perna F. et al., Cancer Cell 2017 (5). We believe that this annotation of antigen protein expression represents a valuable resource to predict potential on-target off-tumor effects and help the selection of a suitable antigen based on the current available list of targets.

## Methods

Data regarding protein target associated effector function, % AML patient expression, and AML cell (blast/LSC) expression were gathered through a systematic review of CAR T-cell AML publications available to our knowledge. Additionally, the summarized list of AML CAR products was made through searching of keywords “AML” with “CAR” or “STAR” in the clinicaltrials.gov public database available on October 7^th^, 2022. All clinical trials available were included. Surface protein expression of each AML target was extracted from the Human Protein Atlas Database, Human Protein Map, and Human Protein Atlas.

## Conclusion

To date, twenty-six AML-associated target antigens have been proposed as targets for CAR T-cell therapy of AML, with many more likely on their way. Through an extensive review of the current literature and the clinicaltrials.gov website, we report the fifteen targets that are currently or have been investigated within a clinical trial with CAR T-cells in AML patients. While the excitement surrounding the advancement of novel AML CAR T-cell antigens is warranted, the validation of many preclinical designs has yet to be completed, and the results of many clinical studies have yet to be published. Thus far, nearly all investigational CAR T-cell products published have shown variable, yet moderate clinical efficacy or significant toxicity. There are many considerations related to this outcome such as the clonal complexity of AML and an evolving toxicity profile as novel CAR T-cell targets enter the clinical landscape. Understanding the AML-specific mechanisms that drive leukemogenesis remains a top priority for the identification of biologically and therapeutically relevant cell surface targets that could be used for curative CAR T-cell therapies tackling the critical mechanisms of leukemia initiation and progression. Leukemia-specific mechanisms such as epigenetic alterations and alternative mRNA splicing that are driven by frequently recurring genetic mutations may expand the CAR T-cell target space that is currently limited in AML. This attractive type of targets that may derive from AML genetic mutations may include post-translational modifications or splice variants of cell surface proteins. Meanwhile, to reduce toxicity and enhance the safety of CAR T-cell therapy targeting leukemia-associated target antigens such as CD33 or CD123 and preventing/predicting on-target/off-tumor effects an appropriate target antigen selection and validation remain critical steps in the development of AML CAR T-cell therapy and that can be integrated within the control of CAR activity by suicide genes or switch-off designs, optimization of CAR construct designs and/or combination approaches. Further, technologies such as deep-scale proteomic analyses with Mass-Spectrometry continue to advance and investigations of protein dysregulation in AML pathogenesis may soon uncover a new list of AML CAR antigens.

## Author contributions

All authors listed have made a substantial, direct, and intellectual contribution to the work and approved it for publication.
